# TEM sample preparation of microsized LiMn_2_O_4_ powder using an ion slicer

**DOI:** 10.1186/s42649-021-00068-5

**Published:** 2021-12-23

**Authors:** Jung Sik Park, Yoon-Jung Kang, Sun Eui Choi, Yong Nam Jo

**Affiliations:** 1 Product Application Support, JEOL Korea, Seoul, 05355 South Korea; 2 Industry University Cooperation Foundation, Hanyang University, Seoul, 04763 South Korea; 3 Korea Electronics Technology Institute, Gyeonggi-do, 13509 South Korea

**Keywords:** Ion slicer, Lithium-ion secondary battery, Backside-ion milling, TEM sample preparation, Broad argon-ion beam (BIB), High-resolution (HR) STEM

## Abstract

The main purpose of this paper is the preparation of transmission electron microscopy (TEM) samples from the microsized powders of lithium-ion secondary batteries. To avoid artefacts during TEM sample preparation, the use of ion slicer milling for thinning and maintaining the intrinsic structure is described. Argon-ion milling techniques have been widely examined to make optimal specimens, thereby making TEM analysis more reliable. In the past few years, the correction of spherical aberration (Cs) in scanning transmission electron microscopy (STEM) has been developing rapidly, which results in direct observation at an atomic level resolution not only at a high acceleration voltage but also at a deaccelerated voltage. In particular, low-kV application has markedly increased, which requires a sufficiently transparent specimen without structural distortion during the sample preparation process. In this study, sample preparation for high-resolution STEM observation is accomplished, and investigations on the crystal integrity are carried out by Cs-corrected STEM.

## Introduction

Lithium-ion secondary batteries comprising a positive electrode, a negative electrode, and an electrolyte have been the most revolutionary materials as a type of rechargeable battery due to their high energy density per unit area. Generally, the negative electrode mainly consists of carbon, which has a theoretical capacity of 372 mAh/g in the fully lithiated state (Shao et al. 2020). To further increase the gravimetric and volumetric capacity, silicon has been studied as one of the most promising negative electrode materials due to its higher theoretical capacity of 3579 mAh/g (Obrovac and Christensen 2004; Key et al. 2009; Miao et al. 2019). Positive electrode materials, including transition metals and lithium-ionic composites containing Li, Mn, Ni, Co, and O, play an important role in determining the performance and cost. For that reason, it is essential to accurately identify the structural information of electrode materials at the atomic level. Although many significant studies for the characterization of lithium-ion batteries have contributed, cutting-edge research showing the structural relationship or behaviour of light elements such as oxygen and lithium has rarely been reported thus far (Pender et al. 2020; Yamada et al. 2013; Miao et al. 2019; Nitta et. 2015). The reason that I point this out is because of the difficulty in preparing a homogeneous TEM specimen that is not structurally distorted. In general, lithium-ion secondary batteries are synthesized in powder form with a size of several to tens of micrometres, and it is not easy to make them uniform structures with a thickness of 100 nm or less. If the powder is 100 nm or less, it can be easily dispersed in a solvent and placed on a grid to be sufficiently transmitted and observed by TEM; however, in the case of secondary batteries, due to size restrictions, the sample must be made thin enough by the cross-sectional preparation to allow electrons to penetrate through the specimen. Generally, the epoxy moulding method has been used for preparing powder samples, but there is a problem in processing because of the inhomogeneous phase/chemical distribution that occurs due to the difference in the milling rate between epoxy and powder (Jiang et al. 2019). The curtaining effect occurs due to thickness variations according to the powder shape; therefore, the condition of the sample in the local area could be severely deteriorated. To overcome these problems, a broad argon-ion beam (BIB) and backside ion milling by means of an ion slicer are introduced (Kato 2004). Current research has focused on the practical aspects of experimental analysis. Due to the problem of beam damage caused by the high acceleration voltage in TEM analysis, the application fields of acceleration voltages of 100 kV or less have greatly increased not only for secondary batteries but also for 2D materials, semiconductor electronic circuit samples micronized to several nanometres, and so on. TEM analysis at a low acceleration voltage has a desirable thickness of 50 nm or less because of electron transmittance and chromatic aberration (Sasaki et al. 2010). Another widely used technique to perform TEM sample preparation is a focused ion beam (FIB), which enables the specimen to be thinned after obtaining cross-sectioned lamella by raster scanning the bulk sample using gallium ions generated from a liquid metal-ion source (LMIS). Basically, it is a scanning electron microscope-based column, so it has a large advantage in targeting the region of interest (ROI) of the sample while observing the electron image; however, artefacts (i.e., sample alterations), such as redeposition, amorphization, vacancies and gallium implantation from the reaction between the heavy gallium ions and sample, are often observed (Basnar et al. 2003; Roediger et al. 2011). In this study, lithium-ion secondary battery powders were preliminarily milled for initial thinning and then finally milled by backside ion milling using an ion slicer. The samples were then evaluated by spherical aberration-corrected scanning transmission electron microscopy (STEM). The ion slicer used a BIB and the surface was protected by the inserted shield belt on the centre of the area to be milled. Thinning was performed by sequentially irradiating both sides with incident ions at a low angle and simultaneously controlling the stage to create a rocking beam. Because it was processed with mechanical polishing down to 100 μm thick (μmt), it could be easily handled while preventing the deformation of the lattice structure caused by mechanical polishing. Notably, conventional ion milling method has been used with mechanical polishing down to 10 μmt or less; however, artefacts often appeared and making the samples difficult to observe by TEM.

## Experimental methods

**Fig. 1:**
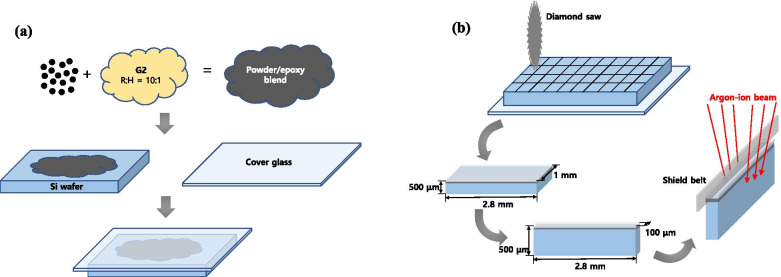
Schematic diagrams showing the sample preparation of a LiMn_2_O_4_ powder and epoxy mixture (**a**) and the conventional method for cutting before ion milling (**b**)

**Fig. 2:**
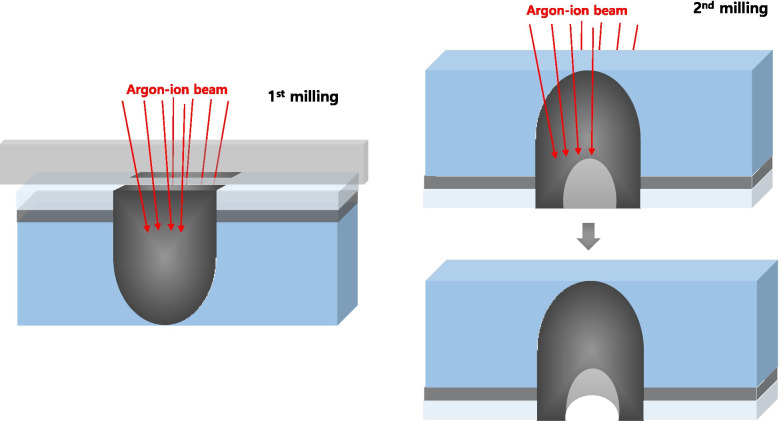
Schematic diagram showing preliminary and backside ion milling with the ion slicer

LiMn_2_O_4_ powders used for composite preparation were supplied by Hanyang University. Figure 1 (a) shows the method for preparing an ion-sliced sample of the LiMn_2_O_4_ powder that is mixed with a G2 epoxy resin/hardener at a ratio of 10:1 to make a powder/epoxy blend. This epoxy mixture is placed on a Si wafer, and then covered with a cover glass. The thickness of the cover glass affects the milling time of the argon beam reaching the resin/powder blend, which is the region of interest, so a 100 μmt cover glass is recommended as appropriate during the secondary operation of backside ion milling. Regarding homogeneous milling, an internal porous structure should be avoided; thus, it is useful to use pressure tongs or decompression devices as a method of removing air bubbles in the blend. Additionally, the sample should be sufficiently hardened on a hot plate for approximately 20-30 min at a temperature of 100 °C. The sample thus obtained is cut into 2.8 mm × 1 mm × 500 μm size using a diamond saw, then, polished until the 1 mm-thick sample becomes the 100 μmt sample with a mechanical polisher using diamond paper (abrasive grit size: 30 μm) as shown in Fig. 1(b). The 100 μmt sample was loaded on the holder stub of the ion slicer (EM-09100IS; JEOL), and argon ion milling is performed after adjusting the shield belt to the centre. Preliminary milling in a large area by BIB is performed for approximately 3 h under the following conditions: the acceleration voltage the incident beam angle are 6 kV and 0.4°, respectively. After that, the specimen holder is turned upside down and mounted to implement backside ion milling at 6 kV and 4° for approximately 1 h, in which ion beam etching is performed until the sample is milled from the bottom of the cover glass to the composite section as shown in Fig. 2. Postmilling is sequentially performed at 3.5° and 3° for 20 min. The final milling step is performed under an acceleration voltage of 1 kV for 10 min. Then, the processed sample is carefully mounted in a reinforcement ring with a diameter of 3 mm. For comparison, sample preparation is performed using FIB (Scios™ DualBeam; FEI), which is the conventional technique for preparing TEM specimens. The sample is coated with carbon as the initial layer to protect the surface from FIB-induced damage and to conduct sample conduction. The carbon-coated layer also provides an excellent amorphous area for aberration tuning. The milling setting is 30 kV and varying beam currents are sequentially used (0.5 nA, 0.3 nA, and 0.1 nA). Finally, the specimen is milled at a low acceleration voltage of 5 kV and a current of 77 pA to remove the damaged layer of the specimen surface. Both samples are evaluated through probe aberration-corrected cold field-emission STEM (JEM-ARM200CF; JEOL) at 200 kV and an energy resolution of 0.3 eV.

## Results and discussion

**Fig. 3:**
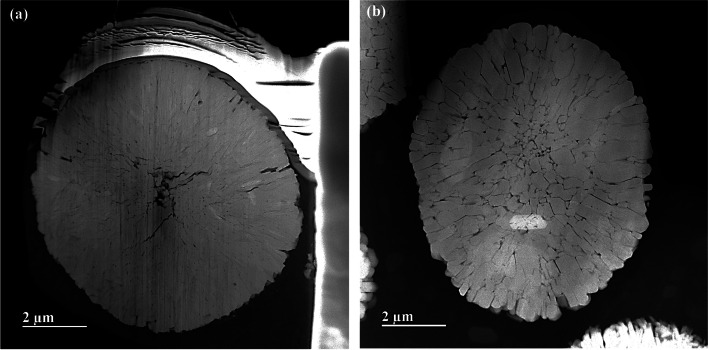
High-angle annular dark field STEM images of the LiMn_2_O_4_ powder which was milled by the focused ion beam (**a**) and ion slicer (**b**)

**Fig. 4:**
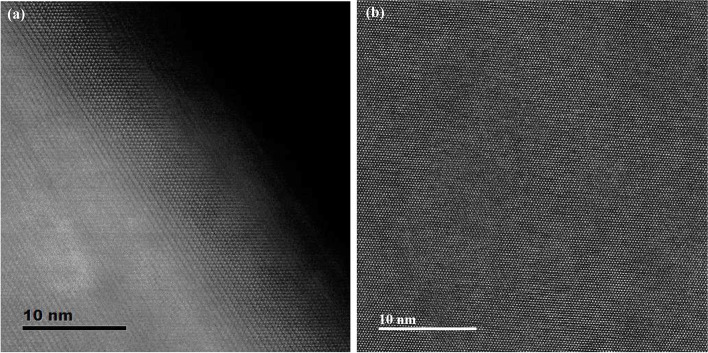
High-resolution HAADF-STEM images showing the edge surface of the LiMn_2_O_4_ powder prepared by FIB milling (**a**) and the inner area of the LiMn_2_O_4_ powder prepared by ion slicer milling (**b**)

**Fig. 5:**
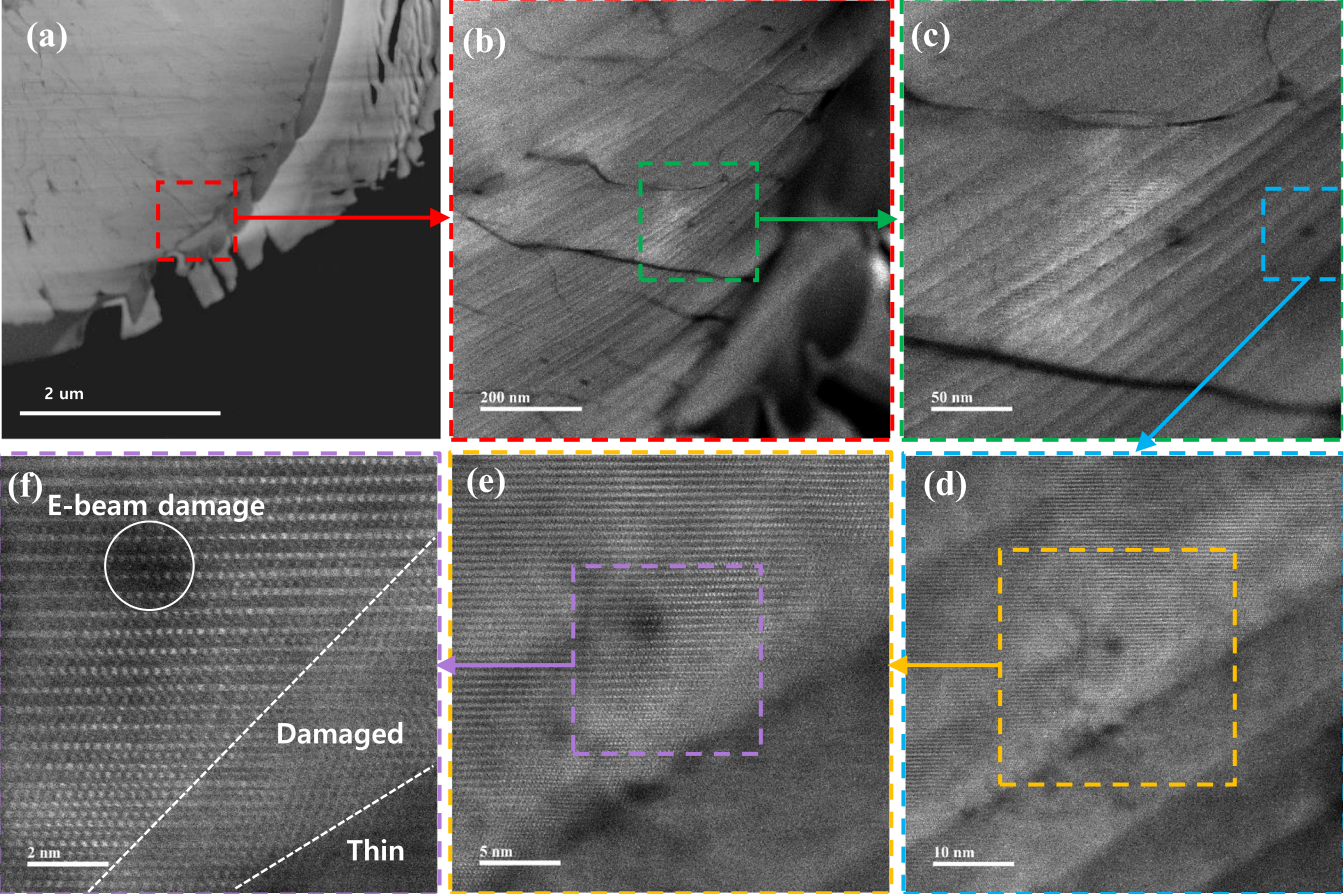
Low-magnification HAADF-STEM image of the FIB specimen (**a**) and magnified HAADF-STEM image of the red- (**b**), green- (**c**), blue- (**d**), orange- (**e**), and yellow-dotted line boxes (**f**); these are respectively magnified at the same location

**Fig. 6:**
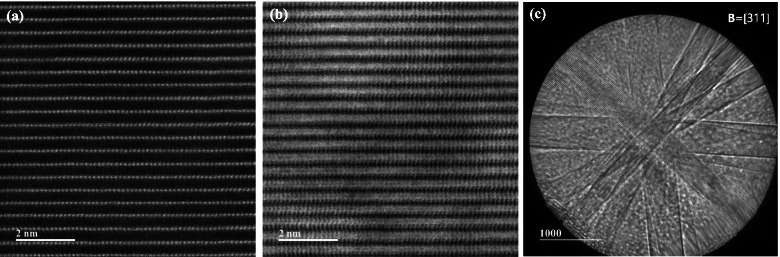
High-resolution HAADF-STEM image (**a**) and annular bright field STEM image (**b**). These images were obtained along the [311] beam direction as shown in the kikuchi image (**c**)

**Fig. 7:**
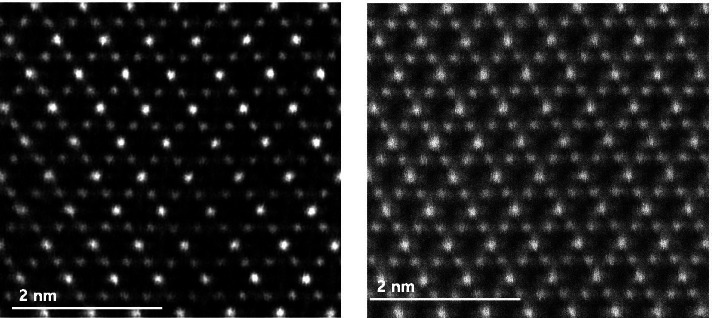
High-resolution HAADF-STEM images of the backside ion milled (left) and conventional ion milled (right) samples

**Fig. 8:**
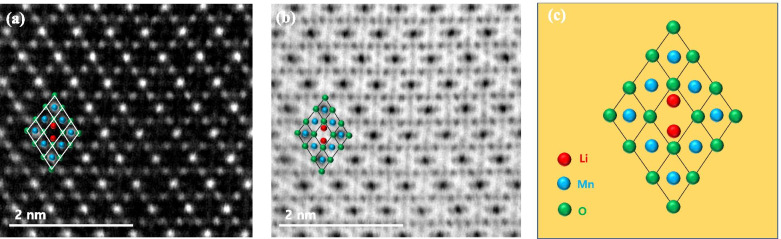
High-resolution STEM image of the ion slicer milled specimen. The atomic arrangement in the HAADF image (**a**) and light elements (Li and O) are clearly seen in the ABF image (**b**). Schematic representation of the LiMn_2_O4 crystal structure (**c**)

**Fig. 9:**
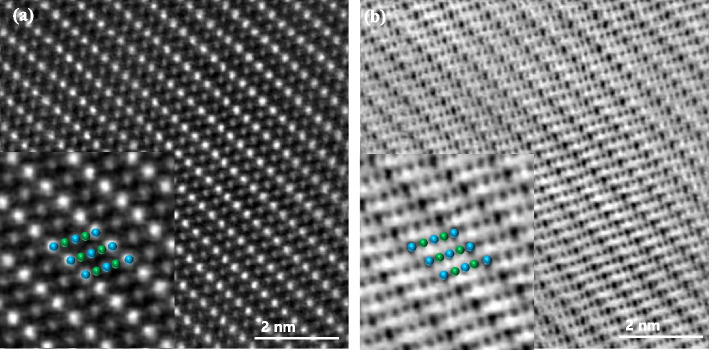
Simultaneously recorded high-resolution HAADF-STEM image (**a**) and ABF image (**b**) of LiMn_2_O_4_ taken along the [010] beam direction. The insets show the structural models of Mn (blue) and O (green)

TEM specimens of the LiMn_2_O_4_ powder prepared by FIB and the ion slicer were evaluated by high-angle annular dark field (HAADF)-STEM. The HAADF-STEM image in Fig. 3(a) shows the curtaining effect generated by the surface roughness of the capping layer in the FIB lamella. In contrast, the HAADF-STEM image of the specimen prepared by the ion slicer using the BIB does not show a curtaining effect inside the cross-sectional area despite the sample having a similar shape, as shown in Fig. 3(b). Figure 4 is the high-resolution HAADF-STEM image for observing the specimen condition at the atomic level. Figure 4(a) shows that the surface side of the powder is milled sufficiently so that the atomic arrangement is clearly observed, but it gradually deteriorates towards the inside because of the variation in thickness, which is caused by the difference in the milling rate of the resin/powder. This has long been considered an obstacle in preparing TEM samples from powders. However, the whole cross-sectional area processed by the BIB technique is clearly observed, as shown in Fig. 4(b). The ion slicer has a shield belt in the centre of the area to be thinned, so it not only protects the surface but also enables the curtaining effect to not occur because the uniformly dispersed argon-ion beam hits the area to be thinned. Consequently, the phenomenon caused by the milling rate difference between the powder and resin is significantly reduced. To evaluate the curtaining effect generated in the FIB sample, high-resolution HAADF-STEM images are obtained at high magnification. Figure 5 sequentially shows the area where the curtaining effect occurs, and the internal atomic structure is observed when magnifying from Fig. 5(a) to (f). The curtaining effect in the form of the striped pattern is observed, as shown in the magnified image in Fig. 5(c). To see further details on the structural information, high-resolution images are obtained, as shown in Fig. 7(d)~(f). The area where the curtaining effect occurs is relatively thin by means of the dark contrast in HAADF imaging. In the high-resolution HAADF-STEM image in Fig. 7(f), the dark spot is artificially created by electron beam damage. The thickness deviations and structural deformation are observed due to the damage caused by the gallium-ion beam. In practice, this phenomenon poses problems for intrinsically interpreting the crystallographic structure. The second set of samples processed using the BIB and beam-rocking technique to avoid the curtaining effect is evaluated, and the results are shown in Fig. 6. The High-resolution HAADF-STEM and annular bright field (ABF)-STEM images are obtained along the [311] beam direction. Based on the results, even though the curtaining effect is significantly reduced, the atomic arrangement is not clearly observed with darker and brighter features in the HAADF-STEM image, especially in the ABF image that includes the phase contrast. To solve this problem, backside ion milling at low voltage is performed as a method of reducing the surface damage and contamination. Then, the conventional method and backside ion milling are compared and evaluated with high-resolution HAADF-STEM. Figure 7 shows that compared to conventional ion milling, backside ion milling results in the structure being more clearly observed; hence, high-precision milling is performed to obtain thinner samples as well as differences in the surface damage and contamination. Furthermore, HAADF- and ABF-STEM images taken along the [110] beam direction are simultaneously obtained to analyse the crystal structure, as shown in Fig. 8(a) and (b), respectively, and the atomic modelling for the LiMn_2_O_4_ crystal structure is illustrated in Fig. 8(c). Due to the Z-contrast, which is the scattering scale that approximates a Z^2^ dependence for the common scattering of the HAADF image, the distribution of Mn, which is a heavy element, can be easily recognized; however, light elements such as Li and O are not observed (Pennycook 1989). Notably, in the ABF image, according to the ABF method with a relatively low atomic number dependence and an electron channelling effect, both atomic columns of relatively heavy elements and light elements can be observed (Findlay et al. 2016). By directly comparing these two images, the column sites of Li and O are understood, and it can be confirmed that these atoms correspond to the atomic model of the LiMn_2_O_4_ structure viewed from the spinel crystallographic orientation. Figure 9 shows the HR-HAADF (a) and HR-ABF (b) images viewed along the [010] beam direction. The oxygen and manganese sites are all clearly visible on the lower left of the cropped images.

## Conclusion

Through this study, we showed that TEM sample preparation using the backside ion milling technique resulted in easy-to-handle samples of microsized LiMn_2_O_4_ powder; notably, TEM samples of this powder are usually hard to handle. It has been confirmed that this technique facilitated thin and uniform TEM specimens, resulting in suitable TEM samples for state-of-the-art aberration-corrected STEM. For precise analysis, it is important to prepare specimens containing the intrinsic state of materials without undesirable artefacts, leading to misinterpretation of the data. This ultrathin TEM sample preparation technique has emerged due to the substantial proportion of trends towards electron microscopy at a low acceleration voltage and electron energy loss spectroscopy (EELS) analysis in the 0-500 eV spectrum region, which contains vibrational (phonon) losses.

## Data Availability

The data and materials described in this paper are available from the corresponding author upon reasonable request.
